# Young-onset behavioural variant frontotemporal dementia mimicking schizophrenia: a case report

**DOI:** 10.1093/omcr/omag181

**Published:** 2026-10-04

**Authors:** Dipta Chandra Kuri, Amrit Lal Das, Subir Chandra Das, Mohammad Saifullah Ahtesam Rana, Md Abdul Alim

**Affiliations:** Department of Neurology, National Institute of Neurosciences & Hospital, Sher-e-Bangla Nagar, Dhaka-1207, Bangladesh; Department of Neurology, National Institute of Neurosciences & Hospital, Sher-e-Bangla Nagar, Dhaka-1207, Bangladesh; Department of Neurology, National Institute of Neurosciences & Hospital, Sher-e-Bangla Nagar, Dhaka-1207, Bangladesh; Department of Neurology, National Institute of Neurosciences & Hospital, Sher-e-Bangla Nagar, Dhaka-1207, Bangladesh; Department of Neurology, National Institute of Neurosciences & Hospital, Sher-e-Bangla Nagar, Dhaka-1207, Bangladesh

**Keywords:** frontotemporal dementia, schizophrenia, diagnostic errors, hydrocephalus, age of onset, case reports

## Abstract

Behavioural variant frontotemporal dementia may initially mimic a primary psychiatric disorder, particularly in younger adults presenting with prominent behavioural and personality changes. We report a 35-year-old man with a three-year history of progressive aggression, social disinhibition, interpersonal dysfunction, emotional withdrawal, aimless self-talking, and later marked reduction in speech. He was initially treated for schizophrenia, but symptoms progressed without meaningful improvement. Routine laboratory investigations were unremarkable. Brain computed tomography was initially interpreted as hydrocephalus; however, neurological review and magnetic resonance imaging demonstrated marked bilateral frontotemporal atrophy with ex vacuo ventricular enlargement. Examination revealed poor cooperation, minimal verbal output, brisk deep tendon reflexes, and frontal release signs, including glabellar tap and snout reflex. Next-generation sequencing and C9orf72 repeat expansion testing were negative. Probable behavioural variant frontotemporal dementia was diagnosed. This case emphasises that progressive, treatment-resistant behavioural decline should prompt reconsideration of a primary psychiatric diagnosis.

## Introduction

Frontotemporal dementia (FTD) comprises clinical syndromes characterised by progressive impairment in behaviour, executive function, or language [1, 2]. It is an important cause of early-onset dementia, although onset before 45 years accounts for only approximately 10% of cases [2]. Behavioural variant frontotemporal dementia (bvFTD), the most common subtype, may closely mimic primary psychiatric disorders [2, 3].

Revised diagnostic criteria identify six core early features: behavioural disinhibition, apathy or inertia, loss of sympathy or empathy, perseverative or compulsive behaviour, hyperorality or dietary change, and a predominantly dysexecutive cognitive profile. Limited insight is common, while functional decline with characteristic frontal and/or anterior temporal abnormalities on neuroimaging supports probable bvFTD [1, 2, 4].

Young-onset dementia is often under-recognised, and atypical behavioural presentations may lead to delayed referral, under-investigation, and misdiagnosis [5]. We report a man with symptom onset at 32 years, initially treated for schizophrenia, in whom progressive behavioural decline, poor treatment response, frontal release signs, and MRI evidence of frontotemporal atrophy supported probable bvFTD.

## Case presentation

A 35-year-old man with 14 years of formal education presented with a three-year history of progressive behavioural and personality change. He gradually became socially withdrawn and emotionally detached. His family reported loss of empathy, social disinhibition, inappropriate language, restlessness, and occasional aggression. These changes resulted in job loss, marital breakdown, and increasing dependence on family supervision. Over time, he developed apathy, self-neglect, poor personal hygiene, aimless self-talking, and markedly reduced speech output during the six months before neurological referral.

Available prescriptions documented amitriptyline 25 mg nightly, followed by clozapine 350 mg/day, aripiprazole 10 mg nightly, controlled-release valproate 300 mg nightly, procyclidine 5 mg three times daily, and lorazepam 1 mg nightly. Exact treatment durations and dose alterations could not be verified and no clinical improvement occurred. There was no history of substance misuse, head trauma, seizures, central nervous system infection, or episodic mood disorder.

Routine blood tests, anti-HIV 1/2 serology, VDRL, thyroid function tests, and vitamin B12 levels were normal. Computed tomography was initially interpreted as hydrocephalus (Fig. 1), prompting neurological referral.

**Figure 1:**
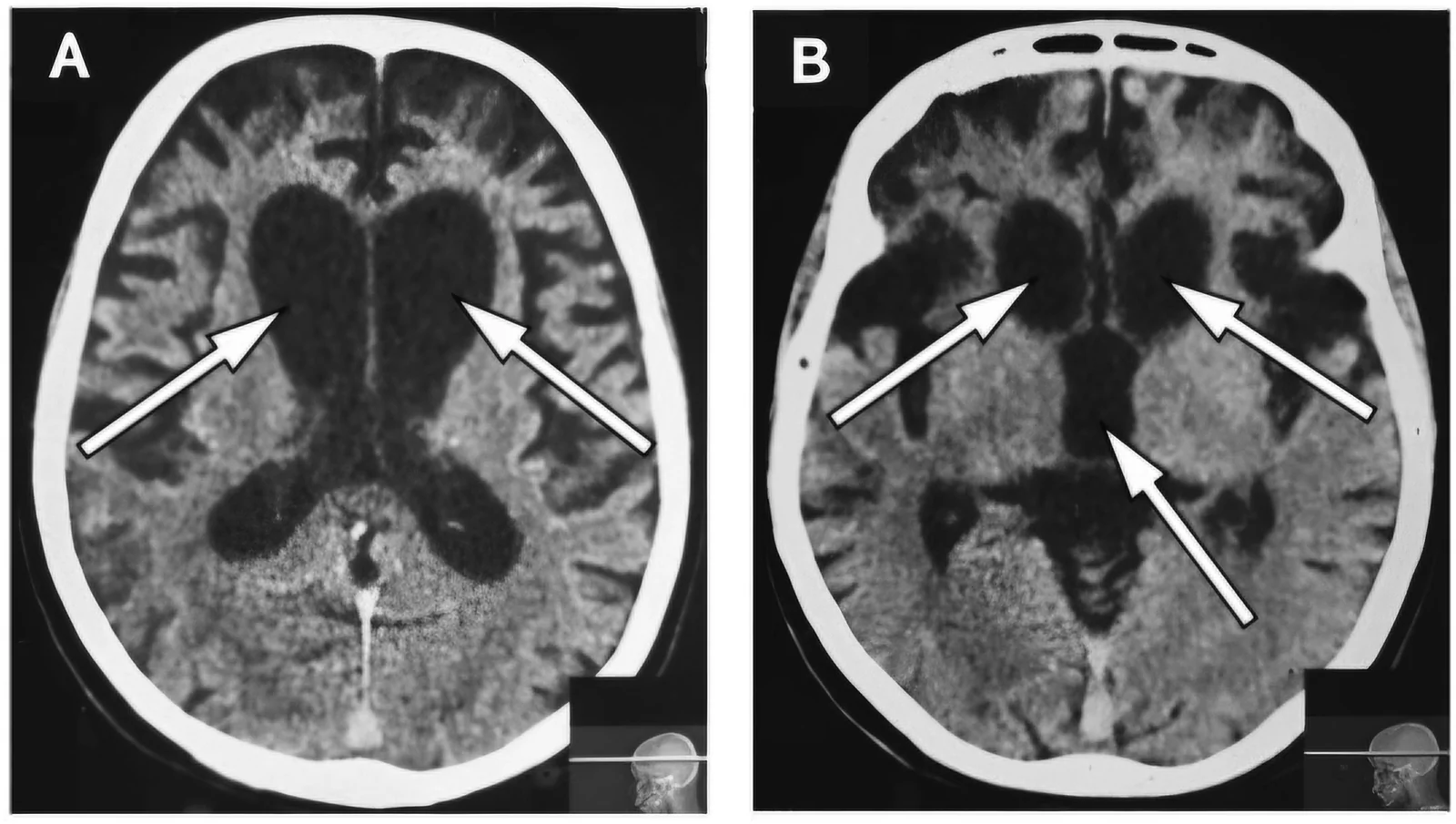
Axial non-contrast CT images at two levels (A and B) demonstrate bilateral frontotemporal-predominant cerebral atrophy, with widening of the cortical sulci and Sylvian fissures. Arrows indicate dilatation of the frontal horns of the lateral ventricles and the third ventricle.

On examination, he was poorly cooperative and minimally verbal. The Mini-Mental State Examination could not be completed because of poor cooperation. Muscle tone was normal, although passive movement elicited gegenhalten (paratonia). Deep tendon reflexes were brisk and plantar responses were flexor. Glabellar tap, snout and grasp reflexes were present, whereas palmomental and rooting reflexes were absent. He walked independently with a slow, normal-based gait and reduced bilateral arm swing, without freezing, festination or overt instability.

Brain MRI demonstrated marked bilateral frontotemporal-predominant cerebral atrophy (GCA score III) with anterior-predominant ex vacuo dilatation of the lateral ventricles. Coronal images showed bilateral medial temporal and hippocampal volume loss (MTA score III). Mild anterior periventricular and deep white-matter hyperintensities (Fazekas score I) were present. FDG-PET and SPECT were unavailable. These findings supported FTD rather than primary hydrocephalus (Fig. 2).

**Figure 2:**
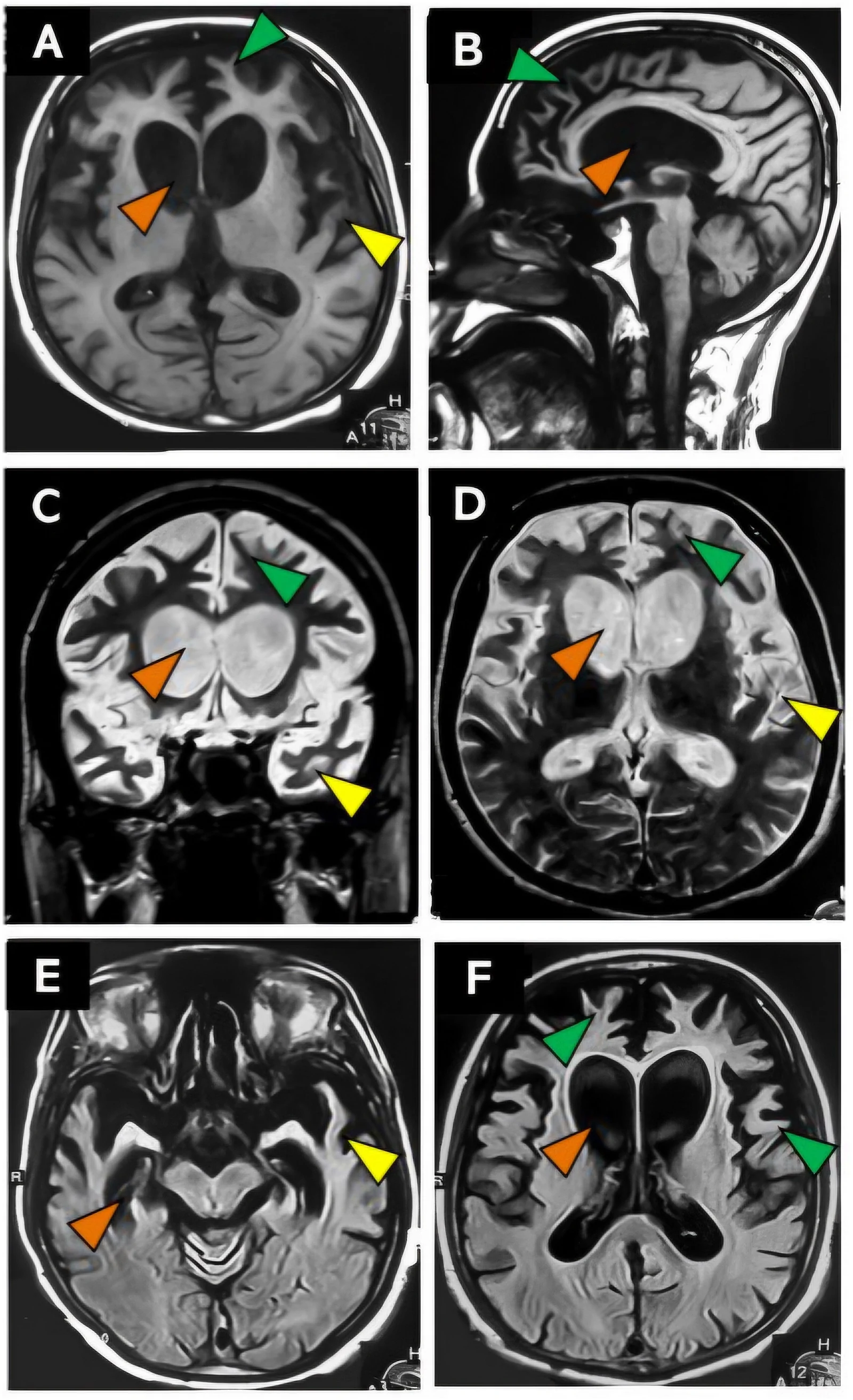
Brain MRI demonstrating frontotemporal-predominant atrophy with ex vacuo ventriculomegaly. Axial T1-weighted (A), sagittal T1-weighted (B), coronal T2-weighted (C), axial T2-weighted (D), and axial FLAIR (E and F) images show bilateral frontal and temporal lobe atrophy with associated sulcal widening and ex vacuo ventricular enlargement. Orange arrowheads indicate frontal horn dilatation (with temporal horn dilatation on FLAIR image E), green arrowheads indicate frontal lobe atrophy and yellow arrowheads indicate temporal lobe atrophy.

Family history was negative for FTD, motor neuron disease, early-onset dementia, and major psychiatric illness. Targeted next-generation sequencing of MAPT, GRN, TARDBP, VCP, CHMP2B, SQSTM1, UBQLN2, and separate C9orf72 repeat-expansion analysis was negative. Based on the progressive behavioural syndrome, functional deterioration, frontal release signs, and supportive MRI findings, probable bvFTD was diagnosed. Management was redirected to supportive care, family counselling, behavioural strategies, and follow-up. Over the next six months, he deteriorated further, with drooling, loss of self-awareness, and worsening self-neglect.

Table 1 summarises the diagnostic revision.

**Table 1:** Clinical course, investigations, and diagnostic revision.

Timeframe	Clinical and diagnostic events
Approximately 3 years before neurological evaluation (age 32 years)	Progressive behavioural and personality changes emerged, including social disinhibition, aggression, inappropriate language, emotional detachment, impaired interpersonal conduct, and functional decline.
During the subsequent 1–2 years	Social withdrawal, loss of empathy, apathy, self-neglect, poor personal hygiene, restlessness, and aimless self-talking became increasingly prominent. He lost his employment, experienced marital breakdown, and became dependent on family supervision. He was diagnosed elsewhere with schizophrenia and received antipsychotic and mood-stabilising treatment without meaningful improvement.
Six months before neurological referral	Speech output became markedly reduced, accompanied by further deterioration in behaviour, self-care, and daily functioning.
Immediately before neurological referral	CT of the brain demonstrated ventricular enlargement, initially interpreted as hydrocephalus, prompting referral for neurological evaluation.
Neurological evaluation (age 35 years)	He was poorly cooperative and minimally verbal, precluding formal neuropsychological assessment. Examination demonstrated brisk deep tendon reflexes, flexor plantar responses, and frontal release signs, including glabellar tap and snout reflexes. Routine laboratory investigations, including thyroid function, vitamin B12, HIV, and VDRL testing, were unremarkable.
MRI and diagnostic revision	MRI demonstrated marked bilateral frontotemporal-predominant atrophy with widened cortical sulci and anterior-predominant ex vacuo dilatation of the lateral ventricles. The ventricular enlargement was attributed to cerebral volume loss rather than primary hydrocephalus. The clinical course and imaging findings supported a revised diagnosis of probable bvFTD.
Genetic evaluation	No relevant family history was identified. Next-generation sequencing of MAPT, GRN, TARDBP, VCP, CHMP2B, SQSTM1, and UBQLN2 detected no pathogenic variant; targeted C9orf72 repeat-expansion testing was also negative.
Six-month follow-up	Progressive deterioration continued, with worsening self-neglect, drooling, loss of self-awareness, increasing dependence, and greater caregiver burden.

**Abbreviations:** bvFTD, behavioural variant frontotemporal dementia; CT, computed tomography; MRI, magnetic resonance imaging.

## Discussion

This case highlights an unusually young-onset presentation of bvFTD that initially mimicked schizophrenia. The patient’s self-talking, social withdrawal, behavioural disturbance, and reduced communication initially supported a psychiatric interpretation. However, the longitudinal history revealed progressive behavioural, functional, and speech decline, supporting a neurodegenerative process rather than primary schizophrenia [1–3].

The overlap between bvFTD and primary psychiatric disorders explains the initial diagnostic direction. Approximately half of patients with bvFTD receive a prior psychiatric diagnosis, with delays of up to 5–6 years from symptom onset [6]. Young-onset dementia further increases this risk [5]. In this patient, the progressive course, poor response to psychiatric treatment, functional decline, and frontal release signs argued against primary schizophrenia. Psychosis-like features should not exclude bvFTD, as delusions, hallucinatory behaviour, and suspiciousness have been reported, although negative psychotic symptoms are more frequent [7].

Neuroimaging resolved the initial concern for hydrocephalus. Although computed tomography initially suggested hydrocephalus, MRI demonstrated frontotemporal-predominant atrophy with ex vacuo ventricular dilatation rather than primary hydrocephalus. This pattern is consistent with ex vacuo ventricular enlargement accompanying FTLD-related atrophy [8].

Early-onset Alzheimer disease (EOAD) was considered because medial temporal atrophy was present. However, hippocampal atrophy alone has limited discriminatory value, as bvFTD typically shows greater frontal atrophy whereas EOAD more often demonstrates parietal-predominant atrophy [9]. The imaging pattern favoured bvFTD. Autoimmune encephalitis was considered unlikely because of the insidious three-year course and absence of seizures. Huntington disease, Wilson disease, and neurodegeneration with brain iron accumulation were also considered but were less likely because the clinical and MRI findings lacked characteristic movement disorders, caudate or basal ganglia abnormalities, or iron deposition.

Genetic testing was performed because of the young onset and psychiatric-mimic presentation. Current recommendations support C9orf72 screening in possible or probable bvFTD with predominant psychiatric symptoms, regardless of family history [6]. The negative targeted panel and C9orf72 analysis did not exclude variants in untested genes. Broader genomic testing was not pursued because of limited availability, cost, and unlikely impact on management.

The progressive behavioural syndrome, early disinhibition, loss of empathy, apathy/inertia, functional decline, and frontotemporal-predominant atrophy fulfilled revised FTDC criteria for probable bvFTD. In the absence of a pathogenic mutation or histopathological confirmation, the diagnosis remained probable rather than definite [4]. Furthermore, recent Bangladeshi dementia surveys include adults aged ≥60 years, leaving young-onset dementia outside current epidemiological data and highlighting a regional diagnostic gap [10].

## Conclusion

This case highlights that bvFTD should be considered in young adults with progressive behavioural and personality changes, particularly when an apparent psychiatric disorder responds poorly to treatment. Longitudinal assessment, neurological examination, and neuroimaging are essential for distinguishing bvFTD from primary psychiatric illness. Early diagnostic reconsideration may reduce delays and facilitate appropriate counselling, supportive care, and follow-up.

## Data Availability

The data supporting the findings of this study are available from the corresponding author upon reasonable request.
